# Proposing a New Conceptual Syndemic Framework for COVID-19 Vaccine Hesitancy: A Narrative Review

**DOI:** 10.3390/ijerph20021561

**Published:** 2023-01-14

**Authors:** Bara’ Abdallah AlShurman, Zahid Ahmad Butt

**Affiliations:** School of Public Health Sciences, University of Waterloo, Waterloo, ON N2L 3G1, Canada

**Keywords:** vaccine hesitancy, COVID-19, syndemics, framework, COVID-19 vaccination

## Abstract

Background: Discussions regarding syndemics have dominated research in recent years. Vaccine hesitancy has also been propelled to the forefront. In this narrative review, we aim to frame a novel syndemic framework to understand the interaction between vaccine hesitancy, COVID-19, and negative health outcomes. Methods: A non-systematic electronic search was conducted in PubMed and Google Scholar. Search criteria were limited to articles published between November 2019 and June 2022. Articles related to the COVID-19 syndemic and vaccine hesitancy were included. Results: Our review revealed that the adherence to COVID-19 regulations—although they were effective in preventing COVID-19 transmission, cases, and deaths—created a dynamically unstable ‘vicious cycle’ between undesirable health, economic, and social outcomes. The “accumulation” of complex stressors decreased individuals’ cognitive flexibility and hindered them from making decisions and getting vaccinated. Furthermore, it increased individuals’ risk of acquiring COVID-19, losing their employment, increasing poverty, and decreasing healthcare utilization. We illustrated how the amalgamation of sociodemographic and contextual factors associated with COVID-19 might impact people’s vaccine decisions, making them more hesitant toward COVID-19 vaccination. Failing to receive vaccinations increases the chances of COVID-19 transmission, hospitalization, and other negative health outcomes. Conclusions: Understanding the interaction between these factors is essential to provide policymakers with inspiration to set appropriate interventions for promoting COVID-19 vaccination acceptance to decrease the overall burden of pandemics.

## 1. Introduction

A syndemic, or synergistic epidemic, is defined as the accumulation of two or more epidemics or disease clusters that are present concurrently or sequentially in a population, and that have biological, social, or environmental interactions that worsen the burden of the disease [1,2,3]. The theory of syndemics was first proposed in the early 1990s to investigate how social, biological, environmental, political, and economic conditions cluster and interact synergistically with other epidemics, enhancing adverse health outcomes and multiplying the burden of disease interaction [1,2,3]. After the emergence of Singer’s syndemic theory (1996), the concept of syndemics and disease interaction has become broadly used and accepted in public health and biomedical fields, epidemiology research, and among healthcare providers and physicians [1,2,4]. Multimorbidity and dual diagnosis have been extensively captured in the last two decades, and the syndemic approach to understanding either disease–behavioural conditions or disease–disease interaction have been documented in the previous literature [4]. Regarding disease–disease interactions, many articles discussed the syndemic interaction between HIV and tuberculosis [5,6], diabetes and depression [7,8], and COVID-19 and non-communicable diseases [9,10]. At the same time, disease–behavioural condition interactions were conceptualized by using lexicalized acronyms and multidimensional syndemic frameworks to understand the clustering of population-level/individual-level health, and its determinants across different dimensions [4]. For instance, the term “VIDDA” was used to illustrate the complex interactions of Violence, Immigration, Depression, Diabetes, and Abuse [11,12]. Other researchers coined the “SAVA” term to characterize a triplex health condition that involves the interaction between Substance Abuse, Violence, and AIDS [13,14]. In the present, these lexicalized acronyms have become widely published, and new frameworks have appeared, such as the “SUMIC” syndemic (i.e., Substance Use, Mental Illness, and Familial Conflict non-negotiation) [15], “SAVID” (i.e., Substance abuse during condomless sex, childhood and adolescent sexual abuse, violence, internalized homonegativity, and depression) [16], and “PHAMILIS” (i.e., Physical Health problems, Abuse, Mental Illness, Loss, Instability, and Substance use) [17,18].

On the other hand, other authors have argued that little attention has been drawn to syndemics from a medical anthropological perspective [12]. Syndemics were viewed by anthropologists as examples of how the concept of co-morbidities affects humans from multidimensional and ecological perspectives, and the ways in which culture and society are organized around or influenced by issues of health, healthcare and related issues [19]. In doing so, they stressed the importance of studying the biosocial processes that reflect how social, cultural, and economic factors influence individual-level experience, instead of only studying disease–disease interaction [12,19].

During the COVID-19 pandemic, much attention has been drawn to syndemics, and discussions regarding disease interactions have dominated research in recent years [20,21,22]. Vulnerability to acquiring COVID-19 was seen as a syndemic, as it increases in the presence of poor social, economic, and environmental factors [23,24,25,26]. These factors, such as social inequality, marginalization, poverty, and injustice, can contribute to COVID-19 clustering, which in turn increases treatment costs [22,23,24,25]. Hundreds of articles demonstrated the intertwined relationship between the COVID-19 pandemic and many adverse health and socio-economic outcomes [24,27]. The outcomes were mainly linked to non-communicable diseases (such as diabetes and cardiovascular disease) [10,28,29], infectious diseases (such as tuberculosis, malaria, and HIV) [29,30,31,32], nutrition insecurity [33,34], mental health inequities [35], climate change, gender-based violence [36], and racism [35,37,38]. A recent rapid review published in 2021 revealed that health emergencies resulting from the COVID-19 pandemic and its associated risk factors (e.g., financial stress, service closure, social distancing, and quarantine) exacerbated gender disparities in terms of income level, work environment, and job security [36]. Consequently, these pandemic-related constraints increased women’s vulnerability to violence and discrimination, and reduced their access to mental health and healthcare services [36]. Another study found that the COVID-19 pandemic complicated the treatment and management of tuberculosis and diabetes due to socioeconomic inequalities and hospital inaccessibility faced by people during the lockdown [29]. Taken together, the COVID-19 pandemic–syndemic acts as the nexus between intensifying health inequalities and worsening the burden of other diseases.

On the other hand, vaccine hesitancy for COVID-19 vaccines has been propelled to the forefront [39,40]. Vaccine hesitancy is defined by the SAGE working group as “the delay in acceptance or refusal of vaccination despite the availability of vaccination services” [41]. Additionally, prior research substantiates the belief that vaccine hesitancy is an attribute ascribed to people who hold an intermediate position along a continuum ranging from full support for vaccination to strong opposition to any vaccine [42,43,44]. Understanding the factors and determinants that influence vaccine hesitancy have become increasingly important, especially during the COVID-19 pandemic, as governments around the globe have been struggling to convince groups of their populations to participate in vaccination protocols [39,40,45,46,47]. In a scoping review published in 2021, the authors investigated and identified significant predictors of COVID-19 vaccination acceptance, refusal, and hesitancy [48]. The findings showed that many demographic, social, and contextual factors were associated with triggering vaccine hesitancy, such as age, gender, education level, race/ethnicity, vaccine safety and effectiveness, influenza vaccination history, health history/medical conditions, and lack of trust [48]. Other previous studies and systematic reviews revealed that different population subgroups may experience greater tendency towards vaccine hesitancy [49,50,51,52,53,54,55,56]. These groups were mainly women, less-educated populations, low-income individuals, black adults, people with comorbidities (e.g., HIV, chronic kidney disease, and substance abuse), and people living in rural or semi-urban settings [49,54,55,57,58,59,60].

Previous studies and reviews were only limited to investigating the factors that influence COVID-19 vaccine hesitancy. None of them explored how social, demographic, and contextual factors can facilitate the interactions between COVID-19 and vaccine hesitancy, and escalate morbidity and mortality, by using a syndemic framework. Therefore, this narrative review has two main objectives: (1) to map and synthesize the literature that captures the COVID-19 syndemic and vaccine hesitancy during the COVID-19 pandemic; and (2) to frame and conceptualize a novel syndemic conceptual framework to understand how different factors interact, resulting in a disparate risk of increasing vaccine hesitancy, COVID-19 transmission, and worsening negative health outcomes.

## 2. Literature Review Search Strategy

This narrative review targeted the existing literature on the COVID-19 syndemic and COVID-19 vaccine hesitancy to identify and summarize what has previously been published. Since this is a narrative review without a strict protocol to be followed, a non-systematic electronic literature search was conducted primarily in health science databases, such as PubMed, using the following MeSH terms and search strategies: ((COVID-19 OR SARS-CoV-2 OR Pandemic*) AND syndemic*) OR ((Vaccine OR Vaccin* OR Immuniz*) AND syndemic*) OR (syndemic*) OR (Vaccine hesitancy AND COVID-19 syndemic*) OR (syndemic* AND interaction*) OR (Syndemic* AND coronavirus AND vaccine acceptan*) OR (SARS-CoV-2 AND vaccine hesitancy AND syndemic*). Google Scholar was also searched to find a broader number of related articles using a plain-language search such as: “Syndemics and COVID-19” or “Syndemics and COVID-19 vaccine hesitancy” or “Syndemics in COVID-19 era”. Search criteria were limited to articles published in English between November 2019 and June 2022. As a result, 104 articles were yielded from the search.

Non-standardized inclusion and exclusion criteria were used. Relevant studies were selected only if they (1) focused on the COVID-19 syndemic and (2) were contextualized to COVID-19 vaccine hesitancy. All relevant studies were reviewed regardless of their types or designs, and all countries of origin were eligible for inclusion. In addition, any relevant articles found outside our search strategy were included if their content related to this review. Articles were excluded if they were (1) not published during the COVID-19 pandemic, (2) irrelevant to either COVID-19 vaccine hesitancy or the COVID-19 syndemic, and (3) non-journal articles. Finally, the included articles (*n* = 40) were peer-reviewed articles, perspectives, reviews, editorials, and commentaries. These articles were reviewed, and relevant data were extracted to report the key findings.

## 3. Key Findings from the Literature

### 3.1. COVID-19 as a Syndemic

Since November 2019, the abrupt, unpredictable, and quickly growing number of COVID-19 cases has resulted in significant and devastating adverse health, economic, and environmental outcomes all over the world [61]. The exacerbating burden of COVID-19 caused a direct impact on people’s health, patient care, hospital capacities, workplaces, country productivity, and financial stability [62]. Based on the COVID-19 level of severity and spread, it was classified as a pandemic by the WHO [63]. At that time, COVID-19 research had recently started, and scientists, public health professionals, medical researchers, and medical anthropologists did not have a deeper knowledge about the unintended consequences of the COVID-19 pandemic. As time went by, seminal contributions were made examining the nature of COVID-19, as well as its cumulative and intertwined characteristics [37,64,65]. From there, research began to be oriented toward the term syndemic, and authors started to drive further development of the idea that COVID-19 can be seen as a syndemic. Miller Singer and his colleagues, Nicola Bulled and Rebecca Irons, were the first medical anthropologists who coined the “COVID-19 syndemic” term, and they discussed and conceived the idea of considering and characterizing COVID-19 as a “syndemic” instead of a “pandemic” [66,67,68]. Irons argued that some researchers and observers stated that COVID-19 affected anyone, regardless of their gender, social class, background, or geographical location; thus, it is a disease that knows no discrimination [68]. However, Irons contradicted this theory and supported her argument by confirming that COVID-19 is inequitably distributed and severely affects elderly people more than younger people, and influences those with underlying health conditions more than healthy people [68]. However, it is no longer right to say that COVID-19 has nothing to do with the discrimination. Hence, many authors supported the notion of reframing COVID-19 as a syndemic instead of a pandemic.

During the same period, Singer and his colleagues shed light on the fact that, even though the COVID-19 pandemic spread globally, multiple countries were impacted by COVID-19 differently based on each country’s resource availability, contexts and demographics, previous pandemics, management plans, mitigation strategies, and healthcare systems [66,67]. As such, South Africa is a focus of attention, as it has been barely affected by the COVID-19 pandemic, besides its rich history of facing past pandemics and diseases, such as HIV/AIDS and the 2009 H1N1 Influenza pandemic [67]. If we look to South Africa as a case study, we can find a multi-faceted problem with extended demographic, environmental, social, contextual, and economic facets. First, according to the WHO statistics, South Africa had the largest number of TB cases, as more than 25% of TB deaths occurred in the African Region [69]. Second, over one-third of people living with HIV in the African Region in 2016 were infected with TB [70]. Third, the problems that faced South Africa during previous pandemics were not only related to the severity and contagiousness of infectious diseases, but also related to poverty, gender inequality, poor health of the workforce, unemployment rates, international labor migration, and violent crime, all of which exacerbated the transmission and co-morbidity of both TB and HIV/AIDS [71,72,73]. All of these diseases together have been involved in portraying the syndemic nature of COVID-19.

However, this is not the whole picture; previous studies have also brought a more comprehensive description of the background of the COVID-19 syndemic, and how COVID-19 restriction measurements could be strongly associated with significant adverse outcomes while interacting with other surrounding social factors [67,74,75,76]. In particular, most low- and middle-income countries that imposed lockdown response strategies similar to those implemented in high-income countries have been adversely impacted by these measures [77]. This is because of their insufficient medical equipment and supplies and limited financial resources. Despite the fact that implementing Non-Pharmaceutical Interventions (NPIs) was effective in preventing COVID-19 cases and deaths in some instances, it still resulted in undesirable health, economic, and social outcomes [77]. These outcomes include physical inactivity, PTSD, high glucose intake/poor diet, domestic violence, and social isolation as a consequence of isolation policies and the long duration of shutdowns [67,75,78]. Moreover, the rigidity of isolation policies, the imposition of longer-duration shutdowns, and the restriction of all non-essential internal movement all resulted in a deterioration of the socioeconomic system, healthcare sector, and business productivity [67,75,78]. Prior to COVID-19, the majority of people living in developing countries were suffering from multidimensional poverty, including income inequalities, healthcare, education, and living standards [79]. When COVID-19 hit these populations, other than its adverse health outcomes, a sharp increase in multidimensional poverty levels was seen [79,80]. The reason behind this phenomenon can be explained by highlighting different situations in different regions globally. For example, harsh governmental restrictions on trade ports and shipments resulted in products’ supply and demand disturbances, insufficient agriculture resources, and food insecurity [33,81]. According to research carried out among developing countries, populations in South Asia, the Middle East, and Sub-Saharan Africa were significantly susceptible to food demand and scarcity, resulting in reduced economic activity and income losses for businesses and workers [81]. Food insecurity due to the COVID-19 pandemic was also described as a multifaceted syndemic that might impact household food insecurity [33]. Consequently, it had an influence on maternal and child nutrition, particularly in families with young children. Previous research has shown that when children do not get enough nourishment throughout their developmental years, it can harm their mental health, which can lead to behavioral issues that hinder their academic performance when they reach school age [33].

Business and school closure were a further two important factors that not only led to increasing unemployment rate and affecting students’ education level, but also triggered unprecedented waves of family violence [82,83]. In Australia, domestic violence and familial abuse have escalated as a result of isolation policies. The authors reported that one cause of this was the loss of social support from extended relatives and neighbors, while others stated that this might be a result of increased alcohol consumption at home when bars and restaurants were closed [84]. Lastly, in the case of“Tighter measures drive non-compliant behaviour”, a study documented that developing countries with more restrictive regulations experienced higher rates of non-compliance to those government regulations [85]. Not surprisingly, the non-compliance rate was associated with labor-insecure individuals’ income. A justification for this is that people with decent jobs, jobs paying low wages, or informal work experienced hardships from stay-at-home orders, which created an adverse reaction and encouraged them to break the rules [85].

The syndemic approach was further expanded to include BIPOC populations (e.g., black, indigenous, and people of color communities), the threat of systematic racism they experienced, and the highest disease burden they suffered during the COVID-19 pandemic [25,86]. Several studies have been conducted to better understand the synergistic effect of numerous illnesses among BIPOC populations during the COVID-19 syndemic, as well as the social, economic, environmental, and political situations that surround them [25,86,87]. BIPOC groups were described as the “More Exposed And Less Protected” populations [88]. Therefore, particular attention should be given to these populations, especially during pandemics and outbreaks. Interestingly, a previous study used the syndemic framework to highlight how healthcare providers’ negligence of patient-related bio- and socioeconomic factors lead to cognitive biases and diagnostic errors/misdiagnosis, which in turn can lead to inadequate treatment [86]. Muhrer et al. demonstrated the idea of disease clustering among BIPOC communities during the COVID-19 pandemic, arguing that physicians and healthcare providers should examine the patient’s social, economic, and environmental aspects—not just biological causes—when diagnosing diseases. Many diseases, such as malaria, diabetes, digestive disorders, respiratory diseases, and cardiovascular diseases, were shown to be more prevalent in low-income neighborhoods than in middle-to-high-income neighborhoods [25,86]. The reason for this is because impoverished populations suffer from poor air quality, malnutrition, inadequate sanitation, and poverty [25,86,87,88]. Therefore, the “accumulation” of multiple and complex stressors left them at a higher risk of acquiring COVID-19. For example, in the U.S., the majority of black people were found to work in low-wage frontline jobs, which put them in a dilemma between staying at home, losing their employment, and becoming unable to pay for their basic needs; or risking COVID-19 exposure and continuing to work [25,87]. Another large concern is their fear of racial discrimination, which creates a mistrust of the healthcare system. As a result, they avoid seeking help from healthcare professionals or visiting hospitals or clinics until they become seriously ill [25,87]. Poor housing, the coexistence of comorbidities, homelessness, and uninsurance issues, all lead black people to be immunocompromised to SARS-CoV-2 infection risk and disease severity [25,86,87].

### 3.2. Vaccine Hesitancy during the COVID-19 Pandemic

Vaccine Hesitancy (VH) was described as a “wicked problem” that is challenging and has no “right or wrong” solution [89]. It is complex, multifactorial, context-specific, and varies over time, communities, and locations. Vaccine hesitancy is not a new phenomenon; widespread skepticism about vaccination has existed for a long time [90]. It first appeared after the development of the first formal vaccine for smallpox in the 1790s [91]. The anti-vaccination movements have been acknowledged as a significant public health threat and a “dangerous trend” that contributed to increasing the prevalence and mortality of vaccine-preventable diseases (e.g., infectious diseases) [92]. Recent vaccination controversies have also emerged, such as those surrounding the association between the measles, mumps, and rubella (MMR) vaccine and the development of autism [93]. As a result, in November 2011, the members of the Strategic Advisory Group of Experts (SAGE) recognized the emerging concerns and reluctance to accept immunization, and established many conceptual models to understand the factors that influence the decision to accept vaccines [94]. Vaccine hesitancy was defined as an intermediate position between full acceptance and full refusal despite the availability of vaccination services [42].

Since the outbreak of coronavirus disease 2019 (COVID-19), vaccine hesitancy has been more recognized, and the anti-vaccination movement has increased at a great rate, which undermines researchers’ efforts to end the pandemic [95]. Global efforts have been made to convince groups of people to participate in vaccination protocols. Vaccine hesitancy during the COVID-19 pandemic has been linked to specific contexts and attributed to a heterogeneous range of determinants, including demographic, social, political, cognitive, environmental, and health factors [96]. Previous research has attempted to understand the complexity of interactions between different factors, vaccine dynamics, and vaccine hesitancy by using a variety of models [42]. The most classic models that have been frequently used in the literature to capture and analyze the factors that affect vaccine hesitancy are the “Health Belief Model”, “Theory of Planned Behaviors”, and “5 C’s model” [97,98,99,100,101]. These models have essentially targeted a multi-factorial approach in order to understand the pathway by which multiple factors are interconnected and exacerbate vaccine hesitancy among different populations [97,98,99,100,101].

A spectrum of determinants of vaccine hesitancy during COVID-19 have been reported in the literature, and they were highly variable [99]. The results from the literature are inconsistent and controversial regarding which group of people are the most hesitant to vaccination. This means that vaccine hesitancy has no stable traits, as it fluctuates across different populations in different regions. In addition, vaccine hesitancy rates vary by country and over time [96]. Interestingly, some researchers point out how people’s opinions and reluctance have changed over the last two years due to the dynamic nature, instability, and uncertainty of the COVID-19 pandemic [101]. Multiple phases of vaccine hesitancy were proposed by Kumar (2022), which are: vaccine eagerness; vaccine ignorance; vaccine resistance; vaccine confidence; vaccine complacency; and vaccine apathy, to explain the variability of people’s behaviors and attitudes toward COVID-19 vaccines [101]. Each of these phases was influenced by a major context and a national measure that was happening at that time. For instance, at the beginning of the pandemic, people were very concerned and afraid of catching COVID-19 and of its complications, so they were very eager to take the vaccine, whereas when vaccines started to be authorized by official governments, people took a step back and were concerned about the fast development of vaccines, which can negatively affect the safety and effectiveness of vaccines. Following this, the rising number of cases and the new emerging contagious variants (e.g., Gamma, Delta, and Omicron) prompted many to reconsider vaccination, especially when the mortality rate decreased in those who were vaccinated. It has been one step forward and two steps back for people to get COVID-19 vaccines; thus, they were stuck in a loop that had no end. For this reason, the term “Hysteresis loop” was suggested to express the negative perceptions experienced by people towards vaccination [102]. This resulted in changes in vaccination trajectory, potentially impeding governments’ efforts to promote vaccine uptake.

Previous research revealed that sociodemographic factors were related to vaccine hesitancy during the COVID-19 pandemic [40,48,103,104]. For example, gender, age, income level, occupation, employment status, religion, education level, race, and ethnicity were all associated with vaccine hesitancy and impacted people’s intention to use COVID-19 vaccines [40,48,103,104,105]. Gender difference was one of the most prominent factors that was linked to vaccine hesitancy during the COVID-19 pandemic [106,107]. There was a consensus in a number of reviews that women were more hesitant to be vaccinated than men due to various barriers and social norms that affect women’s decisions [48,107,108]. Many explanations have been discussed in the literature, such as gender inequality, health disparities, economic insecurity, and poverty [48,107,108]. Likewise, other family members constrain women’s movement, particularly in low-income countries, preventing them from accessing healthcare facilities, including vaccination clinics. The infertility myth was another significant hurdle to vaccination [90,109]. Therefore, the fear of vaccines’ side effects, especially among pregnant women, was frequently reported.

In general, the vaccine hesitancy problem was recurrently linked to unprivileged communities during COVID-19 [110,111,112]. These communities include people who live in low-middle-income countries, BIPOC communities, ethnic minorities, marginalized populations, immigrants, refugees, or even underserved groups who live in high-income countries [110,111,112,113,114]. Past evidence showed that such populations are at higher risk of having poor health and are more likely to catch COVID-19 or be hospitalized due to COVID-19 [110,111,112,113]. In a study conducted by Public Health Ontario in Canada, geographic areas with a high marginalization index were linked to higher levels of ethnic concentration [115]. The authors found that the most ethno-culturally diverse neighborhoods experienced disproportionately higher rates of COVID-19 and related deaths compared to neighborhoods that were less diverse. Additionally, hospitalization and ICU admission rates were four times higher among the most diverse neighborhoods than the least diverse neighborhoods. Similarly, death rates were twice as high in neighborhoods with the most ethnic diversity than in those with less ethnic diversity [115]. Hence, the situation becomes even more problematic when there is low vaccination coverage in these areas because of vaccine hesitancy.

Systematic racism was recognized as a key factor that lies between COVID-19 and vaccine hesitancy [116]. The disproportionate effects of COVID-19, including the unequal distribution of the social determinants of health, lack of transportation, job loss, high mortality rates, and reduced access to healthcare, led underserved populations to distrust unfair health systems, which in turn drove vaccine distrust [113,116]. Black communities in the U.S. are a good example of this; as of July 2021, CDC data indicated that among those who received at least one dose, the number of white people was 1.4 higher than black people [117]; whereas in June 2022, after approximately one year, the percentage of fully vaccinated white people was 5.6 times higher than the percentage of fully vaccinated black people [118]. The growing disparity in vaccination coverage between the two populations is alarming, and raises concerns about vaccine hesitancy and equity in the healthcare system. Vaccine hesitancy linked to health inequities has had a significant impact on other parts of the world [50]. Results from a recent published review revealed that Africa, Eastern Europe, and Central Asia are the most impacted by vaccine hesitancy [50]. Moreover, a higher vaccine hesitancy and refusal rate was observed among Arabic nations, which was related to distrust in the health system and concerns about side effects [119]. Another study by the University of Toronto, Canada, found that immigrants had around two-fold higher risk of COVID-19 vaccine hesitancy than their Canadian-born counterparts. Immigrants also reported higher concerns than non-immigrants on vaccine safety and side effects owing to their distrust of vaccines [120].

Skepticism of vaccine effectiveness, the expedited production of vaccines, low risk perception, fear of vaccine side-effects, parental concerns, and distrust in scientific expertise were other common barriers to COVID-19 vaccine uptake [48]. In addition to this, the problem of the misinformation from the social media “infodemic” is recognized as one of the critical obstacles which stand against providing valid and scientific information to the general population [121]. The notion of politicization, the spread of fake information on social media platforms, and false conspiracy beliefs have all contributed to decreasing vaccine uptake [121]. Health literacy was seen as a core competence to understand basic health information and make decisions about vaccination [122]. This is why people with low health literacy were the most prevalent groups to such fake news, including older adults, minority populations, those with low socioeconomic status, and those with a low education level [122].

It is worth mentioning that the devastating effect of the COVID-19 pandemic itself has increased the level of vaccine hesitancy compared the to pre-COVID-19 era [123]. Familial economic hardship caused by COVID-19 prompted vaccination hesitancy [124]. The majority of those affected by economic difficulty were either vulnerable workers with decent occupations or unskilled individuals working in non-essential activities [125]. As a result, these people encounter a number of logistical problems, such as a lack of money for transportation and inaccessibility to vaccination services [124]. On the other hand, the fluctuation of COVID-19 vaccination policies, the implementation of different measures, mandatory vaccination policies, and the discrepancy between regulations at the national and international levels created confusion and exacerbated hesitation about vaccines in all regions [126]. Furthermore, the poor communication and confusing messages from authority figures impacted people’s confidence in COVID-19 vaccines [126]. It may not only be misinformation which affects people’s decisions to reject vaccines, but also the lack of tools to reformulate the decision-making process and gain a better understanding of the benefit and risks of vaccination.

### 3.3. Framework Development: A Novel Conceptual Framework for COVID-19 Vaccine Hesitancy “COVH”

In order to develop our new syndemic model/framework, we carried out our search, particularly for articles that addressed COVID-19 vaccine hesitancy (e.g., factors affecting vaccine hesitancy, building vaccine confidence, COVID-19 vaccine hesitancy frameworks, COVID-19 vaccine coverage, and factors associated with vaccine uptake) and COVID-19 as a syndemic. Recent syndemic research contains different models and pathways that link multiple disease-exacerbating cofactors to COVID-19 and negative health outcomes. We also reviewed research that focused on applying a syndemic lens to disease outbreaks to fight future public health disasters and ongoing health inequity.

**Figure 1:**
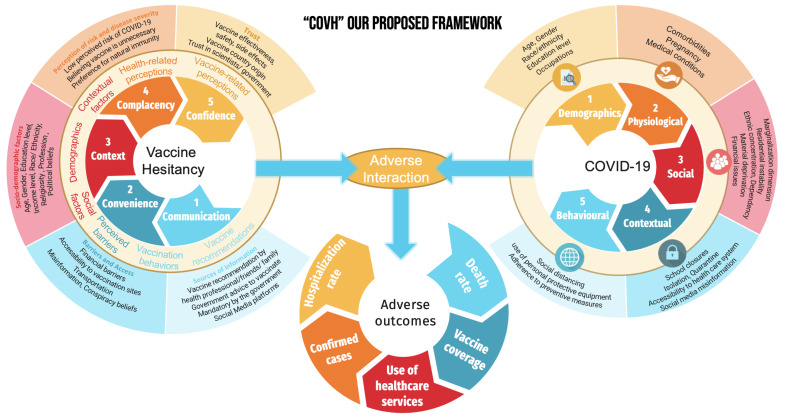
COVID-19 and vaccine hesitancy syndemic framework “COVH”.

Our framework was developed by considering the most commonly discussed factors in the literature, as well as the most prominent previous frameworks. We sought to develop a framework that included three different models: the vaccine hesitancy model, the COVID-19 pandemic model, and the adverse health outcomes model. Furthermore, we aimed to create an extensive and comprehensive model that can (1) be easily communicated, (2) be used by researchers from various disciplines and sectors to combat vaccine hesitancy, and (3) serve as a basic foundation for public health professionals and policy makers to identify underlying health system vulnerabilities. Figure 1 represents our proposed conceptual framework, which illustrates the interaction between vaccine-hesitancy-associated factors and COVID-19-associated factors, resulting in adverse health outcomes.

#### 3.3.1. Components of the “COVH” Syndemic

The first part of the framework includes factors associated with vaccine hesitancy. We used a modified version of the 5 Cs model to investigate the psychological drivers of vaccine hesitancy. The idea of using this specific model is that it covers all the main drivers and barriers to getting vaccinated. The modified 5 Cs model encompasses five categories: confidence, complacency, context, convenience, and communication. Therefore, taking these in turn: first, confidence—do people trust the vaccines, the health system and the authorities more widely? This category comprises factors such as vaccine effectiveness, safety, side effects, vaccine country origin, influenza, vaccination history, and trust in scientists/government. Second, complacency—how do people perceive the potential risks of catching COVID-19? This category consists of factors such as perception of risk and disease severity, low perceived risk of COVID-19, believing the vaccine is unnecessary, and preference for natural immunity. Third, context—how socio-demographic characteristics of people affect their decisions to get vaccines, including age, gender, education level, income level, employment status, region, race/ethnicity, religiosity, marital status, profession, and political beliefs. Fourth, convenience—is it physically and financially easy to get vaccinated, and do people have access to easily understood information about the vaccine in a language that they know? This category includes financial barriers, accessibility to vaccination sites, transportation, and vaccination policies. Lastly, communication—how do people obtain information/recommendations about vaccines, and which sources do they use the most to inform their risk–benefit analysis about vaccination? This category includes vaccine recommendations by health professionals/friends/family, mandatory vaccination by the government, social media platforms, misinformation, and conspiracy beliefs. Overall, all of the previous factors are associated with vaccine hesitancy. Therefore, the different “C’s” are important to tackle vaccine hesitancy for different population groups, and it is important to conduct an analysis of the situation in order to ensure that appropriate interventions are implemented in response. There is no one-size-fits-all approach to this work and that can make it challenging, but it is still essential. We should remember that nobody is safe until everybody is safe.

The second part of the framework includes factors that are associated with COVID-19. These factors can be either demographic, biological, social, contextual, or behavioral. Demographics may include age, gender, race/ethnicity, education level, and occupation. Biological factors may involve comorbidities, pregnancy, and medical conditions, while social factors can be represented by marginalization, poverty, job loss, health disparities, violent crime, food insecurity, and financial issues. Contextual factors can include school closures, isolation, quarantine, access to healthcare, social media, and misinformation. Finally, behavioral factors may be related to social distancing, use of personal protective equipment, and adherence to preventive measures.

The third part of the framework represents the result of the interaction, which can be translated into five major negative health outcomes: death rate, hospitalization rate, vaccine coverage, use of healthcare services, and confirmed cases.

#### 3.3.2. Framing the “COVH” Syndemic

Although vaccine hesitancy (VH) is not a direct mechanism of COVID-19 transmission, it plays a catalytic role in driving the COVID-19 syndemic. Historically, low vaccine coverage pertaining to VH was associated with increased rates of infectious diseases, putting an additional burden on treatment costs [127]. Evidence dating back to 2016 indicates that a 5% decline in MMR (measles, mumps, and rubella) childhood vaccination in the U.S. was associated with a three-fold increase in measles cases, with an additional USD 20,000/case needed for measles treatment, including laboratory investigations, transportation, and vaccination [127].

During the COVID-19 pandemic, VH was attributed to many factors, such as age, gender, vaccine safety, vaccine effectiveness, misinformation, education level, lack of trust, racism, and social inequality, all of which enhanced COVID-19 transmission, morbidity, and mortality [48]. Moreover, COVID-19 has been linked to many adverse health outcomes, such as pneumonia, acute cardiac injury, acute respiratory distress syndrome, and acute liver injury [128]. Taken together, the clustering of these factors resulted in the aggravation of hospitalization rates and Intensive Care Unit (ICU) admissions; thus, subsequent demand for healthcare worker (HCW) staffing and personal protective equipment (PPE) [129,130]. Likewise, the augmentation of treatment prices (e.g., antiviral drugs) and the scarcity of face masks, ventilators, and ICU beds have created shortcomings in the delivery of patient care [129,130]. To date, global statistics show that vaccination rates in low-income countries remain low, and that only 20.9% of the population has received at least one dose of the COVID-19 vaccine [131], with some studies suggesting that VH triggered by misinformation and inequity in vaccine distribution is the driving factor behind such situations [132,133].

Mental health issues can be seen as a salient factor that provoke COVH syndemics, thereby maximizing the cost of treatment of multiple diseases convergence. On one hand, movement restriction and prolonged social isolation during the COVID-19 pandemic affected people’s mental and emotional health [75]. A previous study estimated an additional 53 million cases of mental disorders due to the COVID-19 pandemic globally, which introduced an additional demand to mental health services [134]. Although implementing protective measures (e.g., lockdowns) was effective in preventing COVID-19 transmission, cases, and deaths [135], it resulted in undesirable health, economic, and social outcomes, such as PTSD, high glucose intake, unemployment, domestic violence, and social isolation [67,75]. Vaccination decisions, on the other hand, were perceived as a challenging procedure due to the abundance of infodemics and misinformation on social media [136]. This stressful pandemic and its unprecedented uncertainty weakened people’s cognitive flexibility needed for behavioral adaptation to changing situations, particularly those with low health literacy. This fueled VH, which eventually resulted in not being vaccinated [136]. As a result, unvaccinated people amplified COVID-19 transmission, especially those living in high-density housing [137]. In Canada, unvaccinated adults were 31 times more likely than fully vaccinated adults to be hospitalized due to COVID-19 [138]. Another study discussed how the rates of vaccination coverage and vaccine efficacy influence the number of COVID-19 cases, direct medical costs, death rate, and productivity losses [139]. The interaction between these medical and behavioral conditions has overburdened healthcare infrastructure [129,130]. It also added cost and frustration to patients and their families who either live at a greater distance from healthcare services and cannot afford transportation costs, or those who lost their jobs and health insurance because of COVID-19 [129]. In addition, patients may suffer from other non-monetary and intangible costs, such as productivity loss and decrements in their quality of life.

The systematic discrimination experienced by marginalized populations has established skepticism about the healthcare system and governments, thus triggering VH [25]. Marginalization involves a wide spectrum of demographic indicators, such as race, ethnicity, minority/immigrant status, income level, unemployment rate, education attainment, and residential instability. Such factors make VH even more complex. Higher rates of infectious diseases and poor healthcare utilization among such groups were attributed to longstanding histories of systemic racism, stigmatization, and discrimination [89]. Because of social inequalities, these communities were heavily represented in essential industries, low-income categories, and living in multi-generational overcrowded dwellings, which were seen as “grim reapers” of COVID-19 [89,120]. Furthermore, Indigenous people have experienced centuries of Canadian colonialism and oppression. They were subjected to medical experimentation and vaccine trials in their childhood without their family’s permission [140]. Understandably, such history left scars in their memories and deeply affected their trust in government, making them hesitant to receive vaccines or healthcare.

The disparities in vaccination rates created a disproportionate impact on such populations, leaving them at higher risk of hospitalization and death due to COVID-19 [110]. For example, in Ontario, Canada, hospitalization rates were four times higher among the most ethnically diverse neighborhoods than in the least diverse neighborhoods [115]. More concerningly, if unvaccinated individuals or those who are hesitant to vaccinate have existing co-morbidities (e.g., diabetes, hypertension, or cardiovascular diseases) [141], this may exacerbate their risk of hospitalization. Dong et al. found that the existence of co-morbidities in COVID-19 cases resulted in 21.1% higher hospitalization costs compared to cases without comorbidities [142]. On the other hand, previous research has shown that due to discrimination concerns, marginalized communities tended to avoid seeking help from healthcare facilities until they became seriously ill [143,144]. The delay and avoidance of medical care by people suffering from non-communicable diseases (NCDs) and COVID-19 would create a “double burden” on the healthcare system. Additionally, it would require multiple visits to different clinics and different specialists, with increased responsibilities on HCWs, who would take the brunt of unpaid or underpaid work [8]. The occurrence of syndemics in healthcare systems structured to deliver care on a disease-by-disease basis often leads to insufficient care coordination [8]. Such care fragmentation is commonly linked to undesirable consequences and increasing costs [8,145].

Through our proposed framework, we can illustrate and conceptualize how the sociodemographic and contextual factors associated with the COVID-19 pandemic and vaccine hesitancy can co-occur and interact with each other, resulting in magnified risk of developing adverse health outcomes. For example, according to previous research, immigrants or refugees are among the people who are most likely to encounter discrimination in the labor market, facing substantially higher levels of unemployment and lower wages [146,147]. This is because of the workplace preference to hire native people in higher-skilled jobs or the lower education level of immigrants and refugees, all of which can threaten their food security, accessibility to transport and health literacy. Low health literacy, in turn, could be a direct contributor to obtaining health information from untrustworthy social media platforms and websites.

The amalgamation of these factors can influence this group’s decisions to take vaccines, making them more hesitant toward COVID-19 vaccination. Furthermore, failing to receive vaccinations increases their chances of becoming infected with COVID-19 and being hospitalized. On the other hand, stringent COVID-19 regulations caused the majority of immigrants and refugees to lose their employment, increasing the poverty rate, creating distrust in the healthcare system, and decreasing healthcare utilization. It should be noted that the situation is significantly worse among this sample’s senior population. COVID-19 can exacerbate the symptoms of older persons who already have health problems, putting an added burden on the healthcare system. A lack of transportation can also be a significant barrier to seeking healthcare, resulting in limited healthcare utilization by this portion of the population.

Of further concern is the interactive nature of all the previous factors. As a result of this syndemic interaction, the number of confirmed COVID-19 cases, hospitalization and death rates, and vaccine coverage could all increase. From this example, we can envision how all of these factors in our suggested framework interact in a loop, with each factor being directly or indirectly connected to the others. Furthermore, the proposed pathway implied that some factors may operate as confounders, mediators, or effect modifiers. Hence, the suggested variables can distort the associations between COVID-19, vaccine hesitancy, and adverse health outcomes. Other factors can transmit the effect between multiple variables or affect the direction and/or the strength of the relationship between the three main parts of the framework.

## 4. Discussion

This narrative review aims to provide a novel framework to understand and conceptualize the interaction between vaccine hesitancy and COVID-19, resulting in negative health outcomes. The novelty of this framework comes from its method of engaging with and connecting different factors and determinants that are associated with major public health problems in recent years and linking them to undesirable health effects which can have considerable impacts on individuals and communities. No prior research has used the syndemic perspective to understand how vaccine hesitancy can synergistically interact with COVID-19 and exacerbate negative health outcomes.

Taken together, using a syndemic framework to explore how multiple factors interact with each other in the context of vaccine hesitancy and COVID-19 will provide countries with a guidance tool to set up plans for future pandemic eradication. The real value of the syndemic model lies in its ability to analyze the pandemic by using cross-disciplinary perspectives including biological, psychological, political, environmental, and social interactions to visualize and predict the long-term consequences of the pandemic [20]. For instance, by understanding how determinants of vaccine hesitancy and factors associated with COVID-19 interact and worsen adverse health outcomes, scientists, policymakers, and legislators might be encouraged to change their ways of thinking about responding to future pandemics. Therefore, employing syndemic thinking in building and preparing impactful interventions—at earlier stages—to address accessibility, equity, healthcare setting, mental health, hospitalizations, vaccine hesitancy, official policies, financial issues, and communication platforms can result in effective solutions and long-term investment.

It is worth noting that vaccination is the most globally approved weapon to prevent infectious diseases such as COVID-19. However, a country without a robust vaccine response strategy, appropriate pandemic management, and sufficient vaccination campaigns cannot successfully eradicate viruses or vaccinate a substantial number of its inhabitants [148]. Previous articles have pointed out the significance of having a clear and ready-made deployment and vaccination plan for pandemic preparedness [148,149]. This can be accomplished by (1) conducting a pre- and post-assessment surveillance system, (2) ensuring equity and accessibility to vaccines for all target groups, especially high-risk people and vulnerable populations, (3) creating clear communication tools to inform people about the risks of pandemics and the advantages of vaccinations, (4) maintaining adequate deployment logistics, and (5) increasing vaccination acceptability and raising vaccine awareness. For example, during the 2009 H1N1 influenza pandemic, studies showed that nations with earlier seasonal influenza prevention and control programs, such as the Americas region (AMR), were more prepared for pandemic responses than those without programs, such as low-income countries [148,149,150,151].

### Implications

Taken together, using a syndemic approach to explore how VH and COVID-19 interact will help researchers and healthcare workers to consider ways to combine health services in order to focus not only on individual behavior and diseases but the context within which that behavior/disease occurs, and the emotional, structural, and social demands which drive those individuals and their VH and their risk of acquiring COVID-19. By doing so, we minimize missed opportunities for addressing such interactions more holistically. In addition, our research can inspire policymakers to move upstream and implement impactful interventions—at earlier stages—to address accessibility, equity, mental health issues, and VH in healthcare settings. The findings from this research can result in improving the delivery of healthcare, optimizing patients’ health, and reducing the treatment costs in Ontario as well as Canada.

### Strengths and Limitations

The strength of this study is that, to our knowledge, this is the first review proposing a framework that addresses COVID-19 and vaccine hesitancy. In addition, it highlighted most of the peer-reviewed and non-peer-reviewed articles that discussed COVID-19 as a syndemic, as well as COVID-19 vaccine hesitancy. However, this review still has limitations. First, a non-standardized search strategy and inclusion and exclusion criteria were used. Second, the nature of narrative reviews is considered subjective in terms of selecting which studies to include, analyzing the findings, and drawing conclusions. Third, this review lacks a quality appraisal of the content and the methodologies of the included studies.

## 5. Conclusions

Understanding the interaction between the three parts of our model is essential to providing policymakers and public health researchers with guidelines to set appropriate interventions and good practices to facilitate COVID-19 vaccination acceptance and uptake, as well as COVID-19-associated measures to decrease the overall burden of disease and adverse outcomes. This framework can be a useful contribution to the huge efforts being made to increase COVID-19 vaccination coverage and bringing us towards the end of the pandemic.

## Data Availability

Not applicable.
